# Electrochemical Reduction of CO_2_ in Tubular Flow Cells under Gas–Liquid Taylor Flow

**DOI:** 10.1021/acssuschemeng.2c03038

**Published:** 2022-09-15

**Authors:** Isabell Bagemihl, Chaitanya Bhatraju, J. Ruud van Ommen, Volkert van Steijn

**Affiliations:** Department of Chemical Engineering, Delft University of Technology, Van der Maasweg 9, 2629HZ Delft, The Netherlands

**Keywords:** analytical model, CO_2_ electrolysis, H_2_/CO, mass transfer limitations, slug flow, tubular flow cell, pressure, unit cell approach

## Abstract

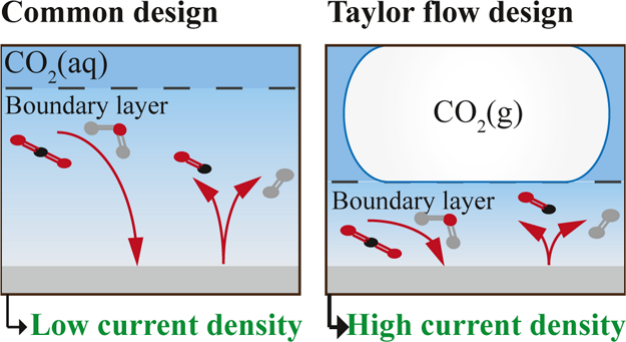

Electrochemical reduction of CO_2_ using renewable energy is a promising avenue for sustainable production of bulk chemicals. However, CO_2_ electrolysis in aqueous systems is severely limited by mass transfer, leading to low reactor performance insufficient for industrial application. This paper shows that structured reactors operated under gas–liquid Taylor flow can overcome these limitations and significantly improve the reactor performance. This is achieved by reducing the boundary layer for mass transfer to the thin liquid film between the CO_2_ bubbles and the electrode. This work aims to understand the relationship between process conditions, mass transfer, and reactor performance by developing an easy-to-use analytical model. We find that the film thickness and the volume ratio of CO_2_/electrolyte fed to the reactor significantly affect the current density and the faradaic efficiency. Additionally, we find industrially relevant performance when operating the reactor at an elevated pressure beyond 5 bar. We compare our predictions with numerical simulations based on the unit cell approach, showing good agreement for a large window of operating parameters, illustrating when the easy-to-use predictive expressions for the current density and faradaic efficiency can be applied.

## Abstract 2

Reactor concepts that enhance mass transfer will enable the electrochemical conversion of various reactants such as CO_2_ to valuable products

## Introduction

Introducing waste CO_2_ as a feedstock for the production of base chemicals can close the carbon cycle and reduce the use of fossil resources.^1,2^ When powered by renewable electricity, the electrochemical conversion of CO_2_ further enables the first steps toward energy transition.^3,4^ The feasibility of this process has been demonstrated at the laboratory scale, notably through impressive advances in catalyst development. However, the main challenge to reach commercialization is imposed by mass transport limitations.^5−7^ A strategy to enhance mass transfer is to actively introduce CO_2_ as a gas flow while keeping diffusion paths between reactants and catalysts short, for example by separating the gas flow from the liquid electrolyte through a gas diffusion electrode.^6,8−11^ However, flooding of, and salt formation in the gas diffusion electrode are common problems and present a major obstacle toward commercialization.^12−15^

**Figure 1:**
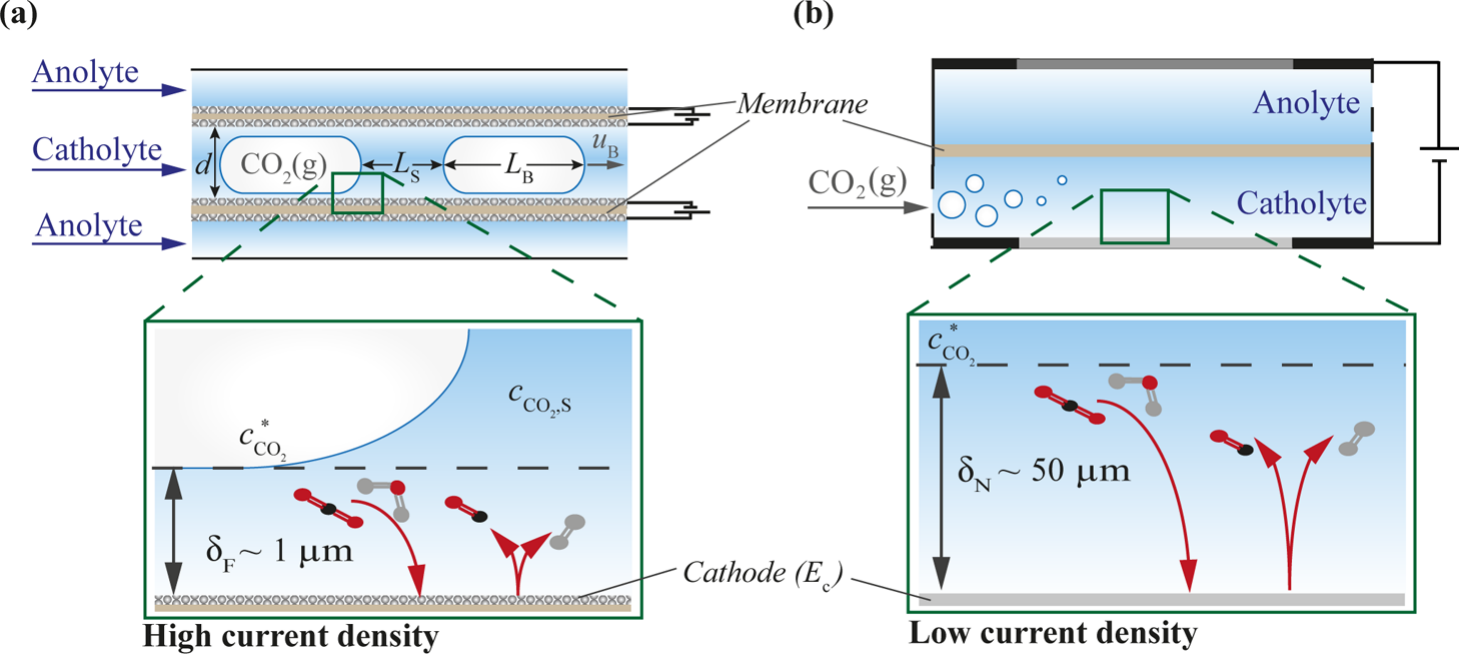
Schematic of a tubular flow cell operated under gas–liquid Taylor flow (a) and an H-cell with static electrolyte and a continuous inflow of gaseous CO_2_ at the cathode chamber (b). Enlarged regions show mass transfer limitations indicated as diffusion layer thicknesses in these systems (film thickness δ_F_ and Nernst diffusion layer thickness δ_N_).

A promising reactor concept that enhances mass transfer without a gas diffusion electrode is a zero-gap membrane reactor operated under gas–liquid Taylor flow.^16,17^ A key feature of this flow type is the thin liquid film between the elongated bubbles and the electrode surface, see Figure 1a. This film is orders of magnitude thinner than the boundary layer for mass transfer in, for example, an H-cell in which CO_2_ is fed by bubbling it through a static electrolyte, see Figure 1b. Reducing this boundary layer to the thin liquid film between the CO_2_ bubbles and the electrode increases the resulting current density by orders of magnitude. While this is a proven concept for heterogeneous catalysis in flow cells with the catalyst coated on the wall,^16−18^ literature on this approach for electrochemical processes is scarce. The first evidence was reported by Zhang et al.,^19,20^ who demonstrated a general increase in activity and selectivity toward CO_2_ in an electrolyzer operated under gas–liquid Taylor flow. Contrary to the literature on heterogeneous catalysis, they primarily attributed the enhancing effect to the mass transfer inside the liquid slugs rather than to the mass transfer inside the thin liquid film around the bubbles. While the experimental demonstration is encouraging, a key step forward is understanding the mechanisms responsible for enhancing mass transfer in electrolyzers operated under Taylor flow. Ideally, the insights are translated into easy-to-use relations between experimental conditions and reactor performance.

In this work, we propose a tubular cell design inspired by the field of fuel cells^21,22^ for CO_2_ electrolysis with a zero-gap membrane electrode assembly and develop a numerical model to reveal how reactor performance in terms of faradaic efficiency and current density is governed by the key features of Taylor flow such as film thickness, bubble velocity, and volume fraction of CO_2_ bubbles over the aqueous electrolyte for a given cathode potential. Based on these insights, we reveal the primary mechanism responsible for mass transfer enhancement and develop easy-to-use analytical relations to evaluate faradaic efficiency and current density for parameters known a priori. We show that these relations are accurate within 10–15% for a wide range of operating parameters by direct comparison to full numerical simulations. For the reader interested in the main results, we structured the paper such that we directly provide the derived easy-to-use analytical relations for the reactor performance in terms of current density and faradaic efficiency, followed by an illustration of the performance for one exemplary system. After that, we present the numerical model and validation. We believe that the easy-to-use analytical relations between operating parameters and reactor performance parameters offer a valuable tool to guide reactor design and experimental studies for electrochemical conversion.

## Summary of Main Results

To illustrate how the operation under Taylor flow enhances the performance of electrochemical reactors, we consider an exemplary system: the reduction of CO_2_ to CO in the tubular cell design shown in Figure 1a, with the inner and outer channels separated by a circular membrane electrode assembly. The liquid catholyte and gaseous CO_2_ bubbles flow through the inner channel as Taylor flow, while the liquid anolyte flows through the outer channel (Figure S1). For simplicity, we consider that CO and H_2_ are the sole two reduction products. In practice, high selectivities toward these products are achieved by preparing the membrane electrode assembly with silver catalyst particles^23,24^ (see Section S1). Therefore, we consider only the mass transfer-limited CO_2_ reduction reaction toward CO and the concentration-independent reduction of water toward H_2_:
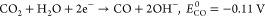
1
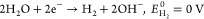
2These reactions are driven by applying a fixed potential (*E*_c_) at the cathode. For simplicity, we consider an electrolyte with a high buffer capacity (e.g., 1 M KHCO_3_), such that local changes in pH can be safely neglected.

The analytical relation between two key reactor performance parameters, the faradaic efficiency and the current density, and operating parameters is introduced for this exemplary system, with all relevant symbols summarized in Table S1. The reactor performance enhancement under Taylor flow is then illustrated for a set of prototypical operating parameters based on the presented analytical relations. This illustration is followed by validating the analytical model against the full numerical model.

### Easy-to-Use Analytical Relations

The faradaic efficiency toward CO (FE_CO_) gives the amount of current driving the desired reduction toward CO over the overall current^25−27^
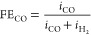
3with *i*_CO_ and  being the current densities for CO and H_2,_ respectively. The current density for a mass transfer-limited species (CO_2_ in the here considered exemplary system) equals^28^
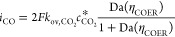
4with Faraday’s coefficient *F*, mass transfer coefficient  under Taylor flow, and the saturation concentration of CO_2_ in the catholyte , which can be determined based on Henry’s law and the Sechenov equation. Additionally, Da(η_COER_) is the Damköhler number for the reduction reaction of CO_2_, which progresses at a rate that depends on the activation overpotential. The activation overpotential η_COER_ is given by the applied cathode potential (*E*_c_) as follows
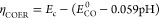
5with the standard electrode potential *E*_CO_^0^ as given in eq 1. The overpotential for the hydrogen evolution reaction (HER) is described similarly with  as given in eq 2. Inspired by the insights developed on heterogeneous catalysis under Taylor flow,^16^ the mass transfer coefficient  for electrochemical conversion of CO_2_ under Taylor flow can be written as
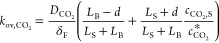
6with  the diffusion coefficient of dissolved CO_2_ in the catholyte, *L*_S_ the length of the slug, *L*_B_ the length of the bubble, and *d* the tubular diameter (see Figure 1a). The film thickness^29^
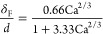
7depends on viscous and capillary forces as captured by the capillary number Ca. The capillary number is defined as  with *u*_B_ being the velocity of the bubbles, μ the viscosity of the catholyte, and γ the interfacial tension between the CO_2_ bubbles and the liquid catholyte. The concentration of CO_2_ in the liquid slugs  is
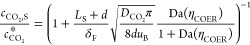
8where the Damköhler number is defined based on the reaction rate of the reduction reaction of CO_2_ to CO (see Section S2). The reaction rate is described by Butler–Volmer kinetics^28^
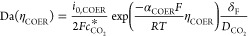
9with  being the inverse of the mass transfer based on film theory, *i*_0,COER_ the exchange current density, α_COER_ the cathodic charge transfer coefficient, *R* the universal gas constant, and *T* the temperature.

The above expressions (eqs 3–9) form a complete set that allows a straightforward calculation of the current density of CO. The current density of H_2_, the species that is considered not to present mass transfer limitations, immediately follows from Butler–Volmer kinetics
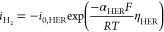
10with the kinetic constants *i*_0,HER_ and α_HER_ and the overpotential η_HER_ (see eq 5). The two key reactor performance parameters, the faradaic efficiency toward CO (FE_CO_) and the current density for CO (*i*_CO_), can now be straightforwardly evaluated using eqs 3–10, in which we introduced the Taylor flow specific behavior in the general electrochemistry framework through the mass transfer coefficient (eq 6) and the concentration in the slugs (eq 8).

### Illustration of Performance Enhancement for Operation under Taylor Flow

**Figure 2:**
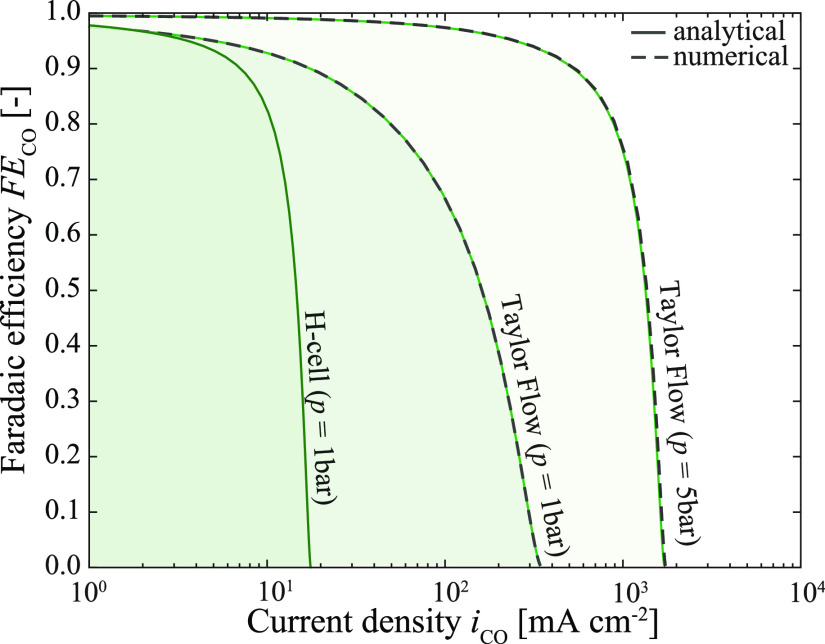
Map of reactor performance as current density and faradaic efficiency toward CO determined by the easy-to-use analytical relations (solid lines) and the full numerical model (dashed lines) for the prototypical operating parameters (*d* = 1 mm, *u*_B_ = 10 mm s^–1^, β_g_ = 0.75) at 1 and 5 bar. The reactor performance for larger *u*_B_, larger *d*, and smaller β_g_ lies under the lines in the green highlighted areas.

To illustrate the reactor performance under Taylor flow, we consider the prototypical operating parameters: CO_2_ bubbles flowing through the central tube with a diameter of *d* = 1 mm at a velocity of *u*_B_ = 10 mm s^–1^, resulting in a thin film around the bubbles with a thickness of δ_F_ = 1.6 μm (see eq 7). We further consider the gaseous CO_2_ bubbles to occupy a fraction β_g_ = *V*_B_/(*V*_B_ + *V*_S_) ≈ (*L*_B_ – *d*/3)/(*L*_B_ + *L*_S_) = 0.75 of the channel volume. The length of the bubble and slug is considered equal to *L*_B_ + *L*_S_ = 5*d*. Using these prototypical operating parameters and the electrochemical/fluid properties listed in Table S2, including the saturation concentration of CO_2_ in a 1 M KHCO_3_ electrolyte at a pressure of 1 bar and ambient temperature ( ≈ 24 mol m^–3^), we calculated FE_CO_ and *i*_CO_ for a range of cathode potentials (*E*_c_ = −0.6 to −3.0 V vs SHE) using eqs 3–10. The chosen potential range allows us to study the reactor performance of the tubular Taylor flow reactor under mass transfer limitations, a regime in which the hydrogen evolution reaction fully overtakes the CO_2_ reduction reaction. The reactor performance of the H-cell can similarly be predicted by assuming (1) film theory for the mass transfer coefficient in eq 4
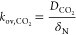
11with δ_N_ ≈ 50 μm^30^ and (2) replacing δ_F_ in eq 9 by δ_N_. Figure 2 shows the predicted reactor performance in terms of FE_CO_ and *i*_CO_ for the H-cell and the tubular Taylor flow reactor, respectively, under ambient pressure for the prototypical operating parameters (solid lines). The *x*-axis intersect of the solid lines directly gives the mass transfer-related reactor performance metric: limiting current density. Comparing this metric for the H-cell and Taylor flow reveals that the reactor performance can be increased by an order of magnitude under Taylor flow. This increase is mainly attributed to the decrease in diffusion layer thickness. We evaluated the reactor performance for the range of operating parameters shown in Table 1. For *u*_B_ and *d* larger and β_g_ smaller than the prototypical operating parameters, the performance lies underneath the solid lines in the green highlighted areas. The reactor performance range can further be increased to compete with industrially required current densities and faradaic efficiencies by operating under elevated pressure, for example, at 5 bar. This increase is understood by the increase in solubility of CO_2_ in the liquid electrolyte ( ≈ 120 mol m^–3^), which enhances the availability of CO_2_ at the electrode.^31−33^

**Table 1:** Range of Operating Parameters Used to Test the Validity of the Easy-To-Use Analytical Relations Against Full Numerical Simulations

symbol	range	unit	label (Figure 3)
*d*	1–3	mm	□ - ◊
*u*_B_	0.01–0.3	m s^–1^	green - black
β_g_	0.25–0.75	−	empty - filled

### Validation of Analytical Solution

**Figure 3:**
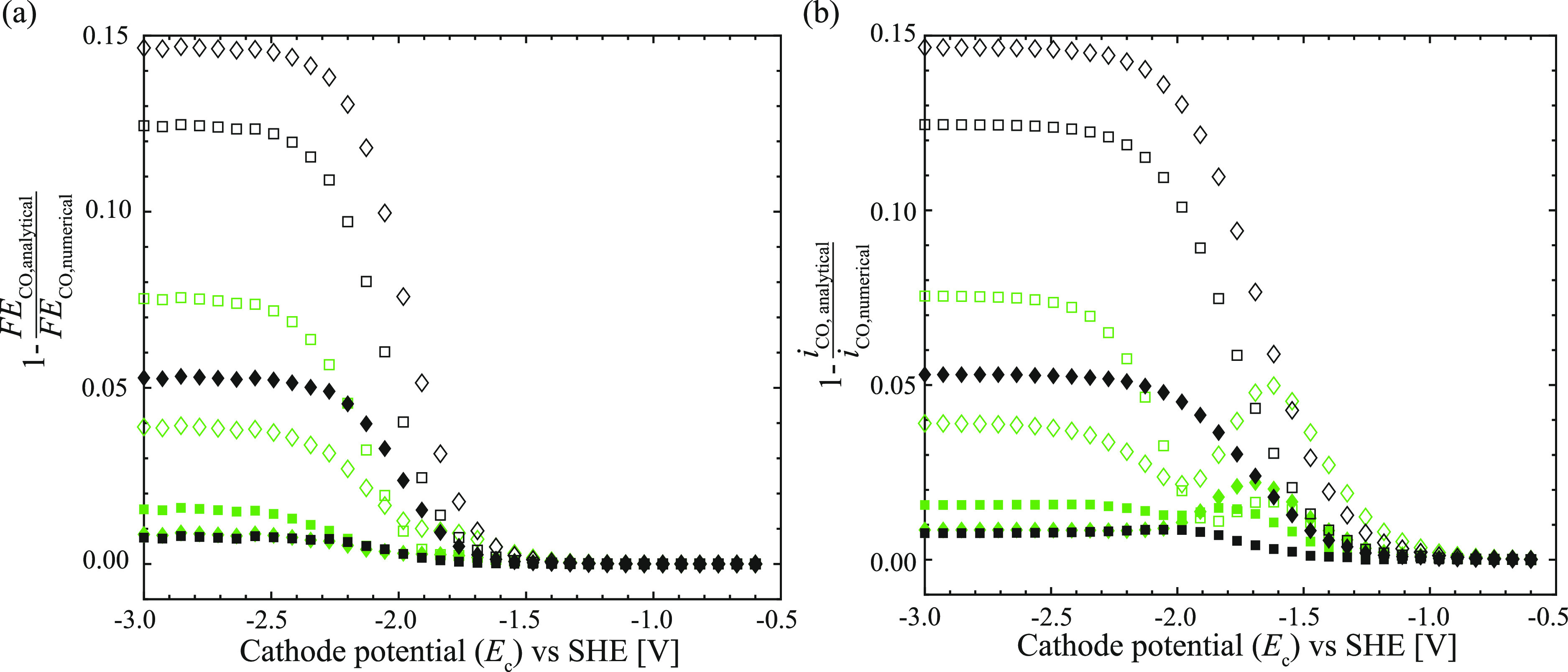
Comparison of the faradaic efficiency (a) and current density (b) calculated with the easy-to-use analytical relations (eqs 3–10) and the full numerical model over the upper and lower bounds of the range of operating parameters listed in Table 1 and electrochemical/fluid properties listed in Table S2. The filled, green squares represent the prototypical operating parameters for Taylor flow at 1 bar, shown in Figure 2; all other symbols are summarized in Table 1.

To test the validity of the easy-to-use analytical relations (eqs 3–10) in predicting the reactor performance for operation under Taylor flow, we compare the prediction of the easy-to-use relations with full numerical simulations. For the prototypical operating parameters, the easy-to-use analytical relations (solid lines in Figure 2) are in excellent agreement with the full numerical simulations (dashed lines). To furhter test the validity of the easy-to-use analytical relations, we consider a wide range of operating parameters (at ambient pressure and temperature), see Table 1. This range is taken from Berčič and Pintar^34^ because the range of capillary diameters overlaps with common channel dimensions for tubular fuel cells.^21^Figure 3 shows that the analytical relations predict the reactor performance within 15% for the studied parameter range. The limit of the analytical model, as derived in Section S3, can be expressed as
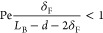
12with the Péclet number Pe, which relates convective to diffusive transport. The limit is met for low bubble velocities (green symbols in Figure 3), which reduces the relative error to less than 8%. It is further evident that the analytical model’s accuracy increases for high void fractions and strongly increases for low bubble velocities (see also Figure S2).

## Full Model and Mechanistic Insights

The numerical modeling of the hydrodynamics and mass transfer of Taylor flow in a unit cell approach^35^ is well documented in the literature.^18,36−39^ Therefore, only this study’s relevant geometrical Taylor flow parameters (Table S3) and assumptions are briefly described here, with all relevant equations and fluid/electrochemical properties summarized in Section S3. The choosen approach allows for a systematic variation of the operating parameters and analysis of the mass transport limitations, providing the necessary mechanistic insights into the contribution of the operating parameters to the reactor performance.

### Full Numerical Model

**Figure 4:**
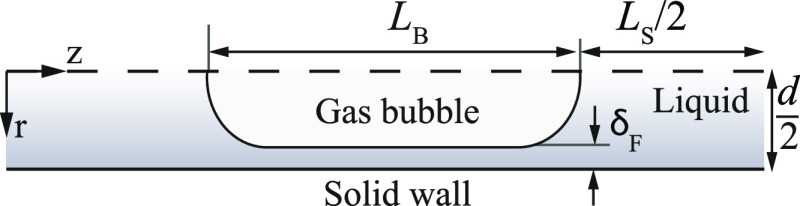
Schematic representation of a two-dimensional axisymmetrical unit cell showing all relevant parameters.

The hydrodynamics and species transport with an electrochemical wall reaction are numerically solved for the cathode compartment in a two-dimensional radial coordinate system following the unit cell approach, as shown in Figure 4. The model is solved steady-state, axisymmetrically, and in the reference frame of the bubble. The dimensionless governing equations are listed in Table S4, with the boundary conditions based on the work by van Baten et al.,^36^ see Table S5. The bubble shape is assumed to be non-deformable^40−43^ (most accurate for Ca ≤ 10^–3^), allowing for the assumption of hemispherical bubble caps connected with a cylinder. In addition, by neglecting viscous effects at the bubble interface, it is sufficient to solely compute the liquid flow with a slip boundary condition at the bubble interface. Equation 7 describes the uniform thickness of the lubrication film between the bubble and the wall. The flow velocities are chosen to fall in the laminar flow regime (Re < 800). The validation of the velocity field can be found in Figure S3.

The dimensionless species balances for CO_2_, H_2_, and CO are listed as convection-diffusion equations in Table S4. Similar to the work by Cao et al.,^43^ the electrochemical reduction reaction is assumed to take place at the wall of the channel and is described by Butler–Volmer kinetics (see boundary conditions in Table S5). Contrary to their study, in which the cathode and anode are placed on the opposite channel sides with the gas bubbles flowing through the inter-electrode gap, the tubular Taylor flow cell is made of a zero-gap membrane electrode assembly (Figure 1a). In this configuration, the cathode and anode are sandwiched together with a membrane in between, minimizing the inter-electrode gap. The CO_2_ bubbles and the gas-evolving products are therefore solely bypassing the cathode, allowing us to neglect the effect of bubbles on the ohmic losses and potential distribution. Additionally, it is assumed that all gaseous products either directly diffuse back into the Taylor bubble or form small bubbles after leaving the electrode, such that local effects of gas evolution can be neglected. Furthermore, it is assumed that the thin catalyst is directly in contact with the anion exchange membrane, and a catholyte with a high buffer capacity (e.g., 1 M KHCO_3_) is used, which leads to ignoring any change in pH and its resulting effect on carbonate cross-over (Section S5 and Figure S4). The validation of the predicted mass transfer coefficient can be found in Table S6.

All two-dimensional unit cell simulations are carried out with the finite element-based software COMSOL Multiphysics 5.6, in which the hydrodynamics are solved decoupled from the species transport. Calculation of the electrolyte-dependent parameters, geometrical properties, and evaluation of the analytical model equations was carried out in MATLAB R2020a. Details regarding the mesh parameters (Table S7 and Figure S5), mesh independence (Figure S6), and solver schemes are given in Section S6.

### Mechanistic Insights into the Mass Transfer under Taylor Flow

**Figure 5:**
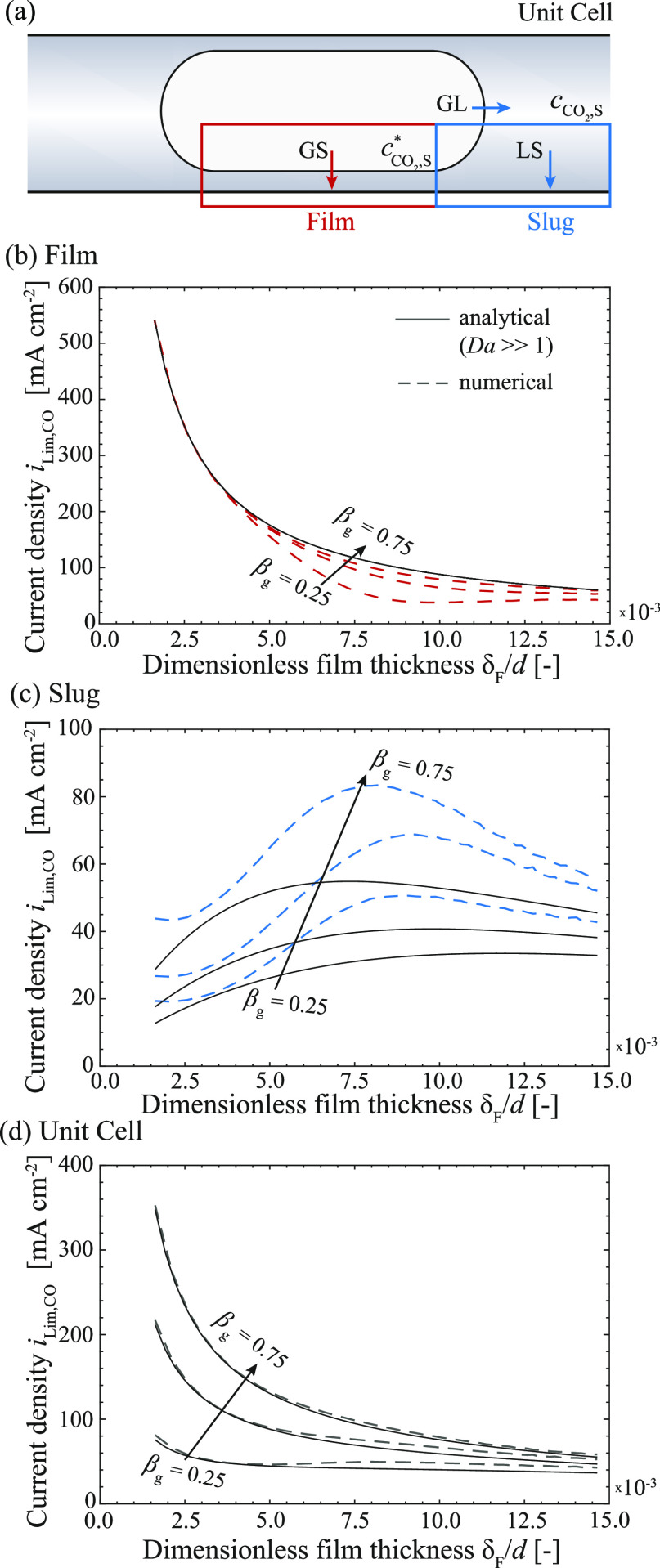
Schematic of the unit cell including the film and slug region with the respective mass transfer routes (a). Limiting current density for a varying film thickness (eq 7) for the film region (b), the slug region (c), and the unit cell (d).

To understand the main mechanism responsible for the enhanced reactor performance under Taylor flow, we now consider the limit in which the mass transfer limitations dominate the reaction kinetics. At this limit, the analytical model is considerably simplified, allowing one to quantify the separate contributions of the film and slug region to the mass transfer coefficient (Figure 5a). This is achieved at high cathode potentials (−3.0 V vs SHE) that translate into high Damköhler numbers. For Da ≫ 1, eq 4 simplifies to the limiting current density equation
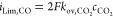
13where we introduce  as the more generic concentration of CO_2_ in the bulk. In the film region,  equals the saturation concentration . In the slug region,  equals the average concentration , which depends on the mass transfer over the caps, as described in eq 8. For high Damköhler, it simplifies to^16^. Considering eq 13, the above expressions for the bulk concentration, together with the mass transfer coefficient based on film theory (eq 11), allows us to straightforwardly see the role of the operating parameters on the limiting current density. While the current density in the film region solely depends on the film thickness, it additionally depends on the void fraction in the slug region.

In Figure 5b,c, the limiting current density *i*_Lim,CO_ calculated with eq 13 and the full numerical simulations are shown for the film and slug region, respectively, for varying dimensionless film thicknesses and three different void fractions. Increasing film thickness generally leads to a decrease in the mass transfer coefficient (see eq 11), which consequently leads to a decrease in current density (see eq 13). This inversely related dependency between the limiting current density and film thickness becomes dominant in the film region (Figure 5b). The numerical simulations further display a slight dependency of limiting current density on void fraction for increasing film thickness, which is not captured by the analytical relation. The deviation between the analytical relation and the numerical model is mostly seen for low void fractions in which the assumption of solely diffusive transport in the liquid film fails (see the limit of analytical assumption in eq 12 and Section S3). The moderate dependency on film thickness and void fraction in the slug region predicted by eq 13 are reasonably in line with the full numerical simulations (Figure 5c). Deviations between the numerical model and analytical relation mostly arise from the simplifying assumption that the diffusion layer thickness in the slug region equals the film thickness (see Figure S7). Importantly, the contribution to the current density from the film region is significantly larger than for the slug region. Only for higher film thickness, the contribution of both regions becomes similar (for additional information, see Figure S6). Figure 5d shows that the current density for the film and slug region together (*i*_Lim,UC_ =  +  and is well captured by the analytical model.

### Reactor Performance under Varying Operating Conditions

**Figure 6:**
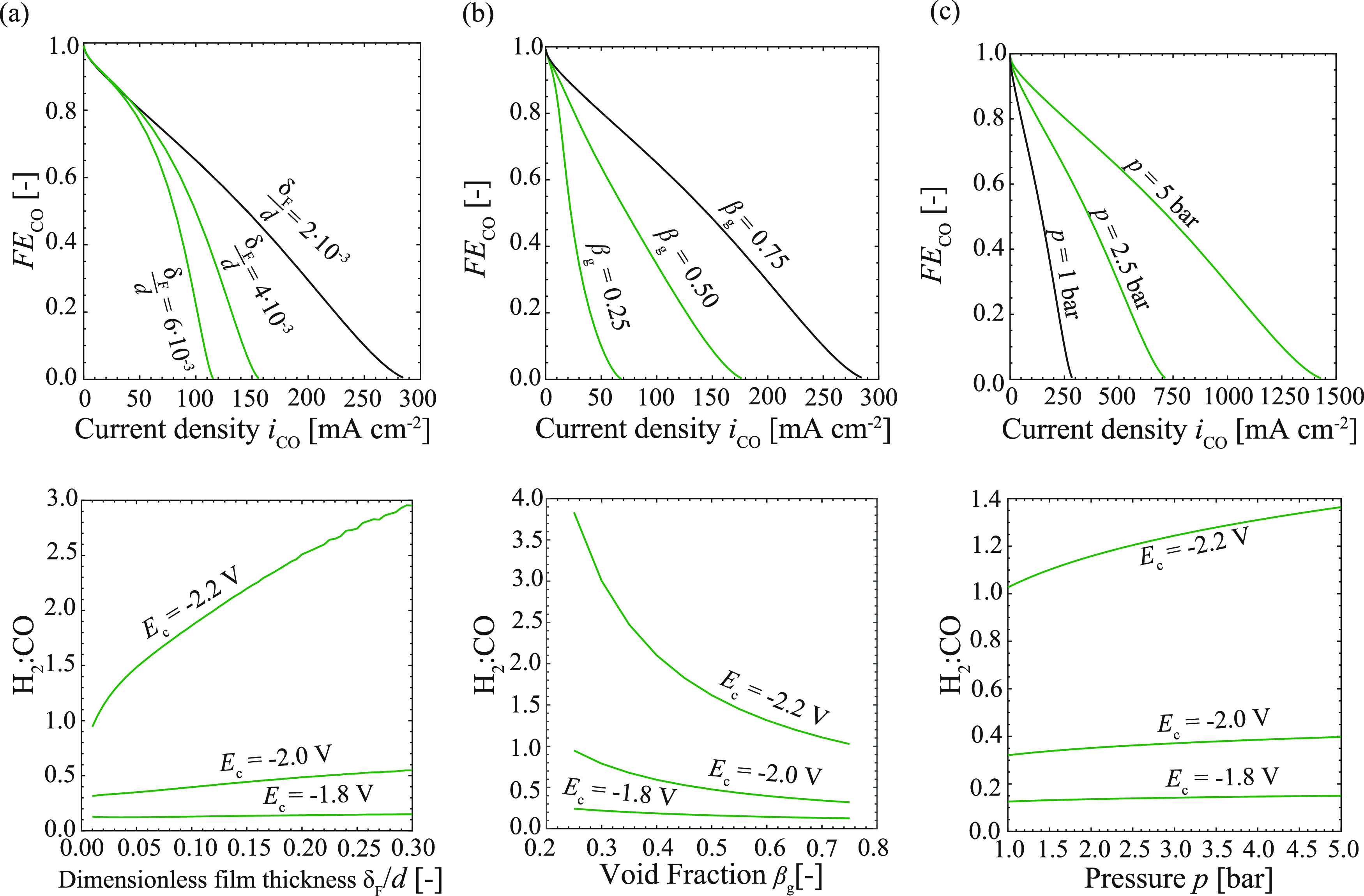
Prediction of reactor performance and the H_2_ to CO ratio with the numerical model for varying film thicknesses (a), void fractions (b), and pressures (c). Black lines in the top figures indicate the same parameters with *d* = 1 mm, *u*_B_ = 14 mm s^–1^, and β_g_ = 0.75 at ambient pressure and temperature.

We quantified the reaction performance in terms of the (limiting) current density and faradaic efficiency toward CO. An additional representation useful in the light of downstream operations is to describe the performance in terms of the ratio between H_2_ and CO_2_ in the produced syngas. Figure 6a shows the influence of bubble velocity in terms of the film thickness (see eq 7) on the faradaic efficiency and the H_2_ to CO ratio for different cathode potentials. Similar to the limiting current density, the faradaic efficiency decreases non-proportionally on increasing the film thickness, leading to a shift in the H_2_ to CO ratio toward the formation of H_2_. This becomes more prominent under higher applied potentials and is explained by increased mass transfer limitations at high cathode potentials. The bubble velocity and therefore film thickness can be directly controlled by the superficial velocities at the inlet^16^ (*u*_B_ ≈ *u*_gas_ + *u*_catholyte_), allowing us to easily control the reactor performance.

The void fraction^16^ can be varied based on the ratio of superficial velocities β_g_ ≈ ϵ_g_ = *u*_gas_/(*u*_gas_ + *u*_catholyte_) and its influence on faradaic efficiency, and the H_2_ to CO ratio is shown in Figure 6b. Higher void fractions result in longer CO_2_ bubbles and shorter electrolyte slugs, leading to an increased film region compared to the slug region. As demonstrated previously in Figure 5, the mass transfer in the film region is an order of magnitude higher than the one in the slug region for low velocities. Therefore, the faradaic efficiency increases proportionally with increasing the void fraction, shifting the H_2_ to CO ratio toward CO.

Apart from varying the superficial velocities, the pressure can be increased to change the saturation concentration of CO_2_ in the liquid electrolyte and thereby increases the faradaic efficiency toward CO (Figure 6c). An increase in the CO_2_ concentration increases the availability of CO_2_ and shifts the equilibrium of the carbon reaction in the electrolyte according to Le Chatelier’s principle, resulting in a slightly more acidic bulk pH. The changes in pH lower the activation potential (eq 5), leading to higher current densities for the same cathode potential at increased pressure^31−33^ (Figure S7). Therefore, the effect of pressure on the H_2_ to CO ratio for the herein shown potentials is comparably lower than the influence of film thickness and void fraction. This implies that pressure is a good approach to increase faradaic efficiencies and reduce the required cell potential, while the superficial velocities present a valuable way to control the H_2_ to CO ratio.

## Discussion and Conclusions

We introduced an easy-to-use analytical model to predict the current density and faradaic efficiency in a tubular flow cell operated under gas–liquid Taylor flow. Comparing the reactor performance to numerical predictions for the electrochemical reduction of CO_2_ to CO shows good agreement within the derived limits. Furthermore, we showed that the limiting current density increases by an order of magnitude for the tubular Taylor flow cell compared to an H-cell reactor. The film thickness and void fraction significantly influence the faradaic efficiency and the H_2_ to CO ratio. Furthermore, the bubble region mainly contributes to mass transfer for low velocities, suggesting a preference for thin films and high void fractions, while for increasing velocities, the mass transfer becomes region independent. The tubular Taylor flow cell architecture offers a design which can be straightforwardly operated under elevated pressure,^44−47^ further improving mass transfer to achieve a high faradaic efficiency (>90%) at a current density of up to 500 mA cm^–2^, demonstrating the general potential of this reactor concept to overcome mass transfer limitations in the field of electrolysis. With tubular flow cells operated under gas–liquid Taylor flow yet to be explored experimentally in the field of electrolysis, we expect the generic insights into mass transfer and the simple analytical model to provide guidelines for experimental studies and reactor design choices.

## Supporting Information Available

Detailed description including the derivation of limits for modeling assumptions and validation of the modeling, all material and operating parameters, current density, and faradaic efficiency for different partial pressures of CO_2_ (PDF)
