# Supplementary material for: Electrochemical Reduction of CO2 in Tubular Flow Cells under Gas–Liquid Taylor Flow
Source: ACS Sustain Chem Eng. 2022 Sep 15;10(38):12580–7. doi: 10.1021/acssuschemeng.2c03038 (PMC9516770; doi:10.1021/acssuschemeng.2c03038)
Supplement: Supplementary file 1 — sc2c03038_si_001.pdf [file sc2c03038_si_001.pdf]

Supporting Information

Electrochemical Reduction of CO_2 in Tubular Flow Cells under Gas-Liquid Taylor Flow

Isabell Bagemihl[1,*], Chaitanya Bhatraju[1], J. Ruud van Ommen[1] and Volkert van Steijn[1]

[1]Department of Chemical Engineering, Delft University of Technology, Van der Maasweg 9,2629HZ Delft, The Netherlands
[*]Corresponding author: i.bagemihl@tudelft.nl

This file includes:
Total - 16 pages, 8 figures, and 7 tables
Figure S1-S7, Table S1-S8

Table S1: List of symbols.

Symbol	Description	Unit
Latin letters		
A_{GL}	Exchange area for gas-liquid mass transfer	$\mathrm{m^2}$
A_{LS}	Exchange area for liquid-solid mass transfer	$\mathrm{m^2}$
Ca	Capillary number	-
$c_{\mathrm{CO_2}}$	Concentration of CO_2	$\mathrm{mol\,m^{-3}}$
$c^*_{\mathrm{CO_2}}$	Saturation concentration of CO_2	$\mathrm{mol\,m^{-3}}$
$c_{\mathrm{CO_2,S}}$	Concentration of CO_2 in the liquid slug	$\mathrm{mol\,m^{-3}}$
c_{wall}	Concentration at the wall	$\mathrm{mol\,m^{-3}}$
Da	Damköhler number	-
D_{i}	Diffusion coefficient	$\mathrm{m^2\,s^{-1}}$
d	Diameter	m
d_{b}	Bubble diameter	m
E^0_{i}	Standard potential	V
E_{C}	Cathode potential	V
F	Faraday constant	$\mathrm{A\,s\,mol^{-1}}$
FE_{CO}	Faradaic efficiency towards CO	V
H^{cp}	Henry coefficient	$\mathrm{mol\,m^{-3}\,Pa^{-1}}$
i_{i}	Exchange current density	$\mathrm{A\,m^{-2}}$
J_{GL}	Gas-liquid molar flux	$\mathrm{mol/m^2/s}$
J_{LS}	Liquid-solid molar flux	$\mathrm{mol/m^2/s}$
k_{f}	Forward reaction rate constant	$\mathrm{mol^{-1}\,s}$
k_{GL}	Gas-liquid mass transfer coefficient	mol/s]
k_{LS}	Liquid-solid mass transfer coefficient	mol/s
$k_{\mathrm{ov,CO_2}}$	Overall mass transfer coefficient of CO_2	mol/s
k_{r}	Backward reaction rate constant	$\mathrm{s^{-1}}$
L_{B}	Bubble length	m
L_{F}	Film length	m
L_{S}	Slug length	m
L_{UC}	Unit cell length	m
p	Pressure	bar
Pe	Peclet number	-
u_{B}	Bubble velocity	$\mathrm{m\,s^{-1}}$
u_{TP}	Two-phase velocity	$\mathrm{m\,s^{-1}}$
Greek letters		
α_{COER}	Transfer coefficient of the CO_2 reduction reaction	-
α_{HER}	Transfer coefficient of the hydrogen evolution reaction	-
β_{g}	Void fraction	-
δ_{F}	Film thickness	m
δ_{N}	Nernst diffusion layer thickness	m
η_{COER}	Activation overpotential of the CO_2 reduction reaction	V
η_{HER}	Activation overpotential of the hydrogen evolution reaction	V
γ	Interfacial tension	$\mathrm{N\,m^{-1}}$
μ	Dynamic viscosity	$\mathrm{Pa\,s}$
ρ	Density	$\mathrm{kg\,m^{-3}}$

1 Cell design

In H-cell configurations, the electrodes are commonly placed with some distance to the membrane, which allows to use either two dimensional electrodes like foils or porous three dimensional electrodes like meshes, felts and papers [1]. In the tubular design the anode and cathode are sandwiched together with a thin polymeric membrane in a zero-gap membrane electrode assembly (MEA) [2]. In this configuration porous electrodes have to be used to allow for the transport of ions and reactants to the catalyst sites. The electrode can then either be made entirely out of the catalyst material, e.g silver meshes or foams or the catalyst material is deposited on an electrical conductive substrate, e.g carbon paper. Several deposition techniques are available while drop-casting, airbrushing and sputtering are widely applied in the field of CO_2 electrolysis [3]. Due to the weak interaction between substrate and catalyst layer these techniques are only limited suitable for tubular flow cells. Therefore, techniques like atomic layer deposition have been proposed in literature for these designs [4]. These techniques can be used to form very thin catalyst layers ($\approx 10\,\text{nm}$)[4, 5].

The cathode in the H-cell could be made of a silver foil, while the cathode in the tubular configuration could be either a silver mesh or a porous transport layer which is coated with silver via atomic layer deposition (ALD). Based on the resulting thin catalyst layer the electrochemical reaction can be assumed to occur at the interface leading to the boundary conditions for the numerical model (Table S5). Additionally, to reduce mass transfer limitations for the reactant CO_2 the catalyst layer would be placed close to the bubble interface and the thickness of the supporting substrate minimized to reduce additional ohmic resistance from the transport of ions to the catalyst side (see Figure S1).

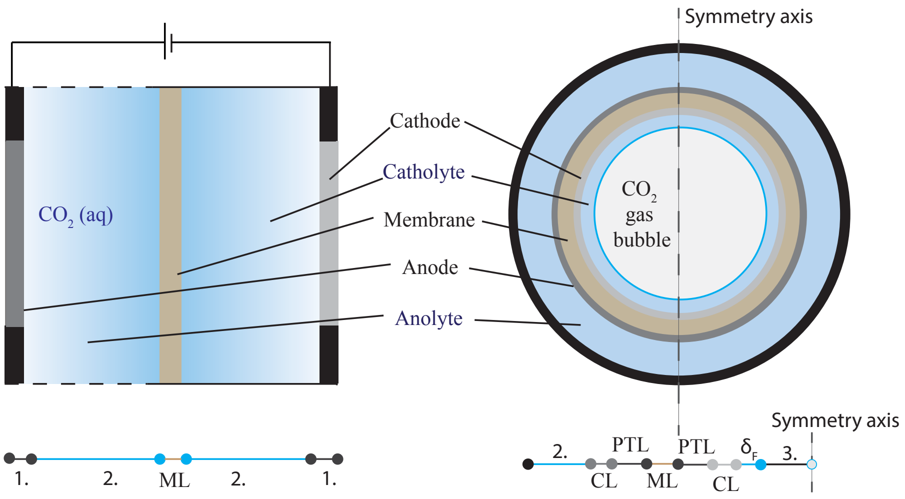

Figure S1: Cross sectional view of the H-cell and tubular Taylor flow configuration with the different layers electrode(1.), electrolyte (2.), membrane layer (ML), catalyst layer (CL), porous transport layer (PTL), film thickness (δ_F) and gaseous Taylor bubble (3.) represented by the line beneath each configuration.

2 Reaction rate dependent concentration in the liquid slugs

The mass transfer over the bubble caps (J_{GL}) depends on the concentration difference between the saturation concentration at the gas-liquid interface ($c^*_{CO_2}$) and the concentration in the liquid slugs ($c_{CO_2,S}$) which is assumed well-mixed, the area of the spherical caps (A_{GL}), and the mass transfer with the mass transfer coefficient k_{GL} described by penetration theory as follows [6]

$$J_{GL} = k_{GL}A_{GL}\left(c^*_{CO_2} - c_{CO_2,S}\right) = 2\cdot 2\sqrt{\frac{D_{CO_2}d}{\pi 2 u_{TP}}}\frac{1}{2}\pi d^2\left(c^*_{CO_2} - c_{CO_2,S}\right), \tag{S1}$$

with the two-phase velocity u_{TP}. The mass transfer (J_{LS}) from the slugs to the channel wall is given by [6]

$$J_{LS} = k_{LS}A_{LS}\left(c_{CO_2,S} - c_{wall}\right) = \frac{D_{CO_2}}{\delta_F}(L_S + d)\pi d\left(c_{CO_2,S} - c_{wall}\right), \tag{S2}$$

with the mass transfer coefficient k_{LS} described by film theory, and A_{LS} for the circular slug region. Equating the fluxes in Eq. S1 and S2, and using the reaction diffusion boundary condition that translates into $c_{wall} = c^*_{CO_2}\cdot 1/\left(1 + Da(\eta_{COER})\right)$, we find

$$k_{LS}A_{LS}\left(c_{CO_2,S} - c^*_{CO_2}\frac{1}{1 + Da(\eta_{COER})}\right) = k_{GL}A_{GL}\left(c^*_{CO_2} - c_{CO_2,S}\right). \tag{S3}$$

Rearranging this equation the ratio of slug concentration over saturation concentration is

$$\frac{c_{CO_2,S}}{c^*_{CO_2}} = \left(1 + \frac{L_S + d}{\delta_F}\sqrt{\frac{D_{CO_2}\pi}{8du_B}}\frac{Da(\eta_{COER})}{1 + Da(\eta_{COER})}\right)^{-1}. \tag{S4}$$

3 Limit of the analytical model

The analytical model, as described in the Section "Easy-to-use analytical relations", is based on a stagnant film approach which allows assuming film theory for the mass transfer in the bubble region and implies that the boundary layer for mass transfer equals the film thickness ($\delta = \delta_F$). This assumption is only valid if the convective transport of species in the liquid film is smaller than diffusive transport. Starting from the stationary convection-diffusion equation

$$0 = D\frac{\partial^2 c}{\partial r^2} - u_B\frac{\partial c}{\partial z}, \tag{S5}$$

with the convective ($u_B \partial c/\partial z$) and diffusive ($D\partial^2 c/\partial r^2$) transport terms, using δ as the characteristic length scale in the radial direction and L_f as the characteristic length scale in the axial direction, we find that the diffusive contribution dominates over the convective contribution when

$$\frac{u_B\delta}{D}\frac{\delta}{L_F} < 1. \tag{S6}$$

With the characteristic length δ Eq. S6 can be rewritten in terms of the Peclét number $Pe = u_B\delta/D$. To arrive at Eq. 12 the length of the liquid film is given as the length of the cylindrical part of the bubble without considering the bubble caps $L_F = L_B - d - 2\delta_F$. It should also be noted that the numerical model does not consider a change in bubble shape, which is expected with increasing velocities. Increasing velocities leads to a flattening out of the rear back of the bubble [6], which can lower the convective transport in the film.

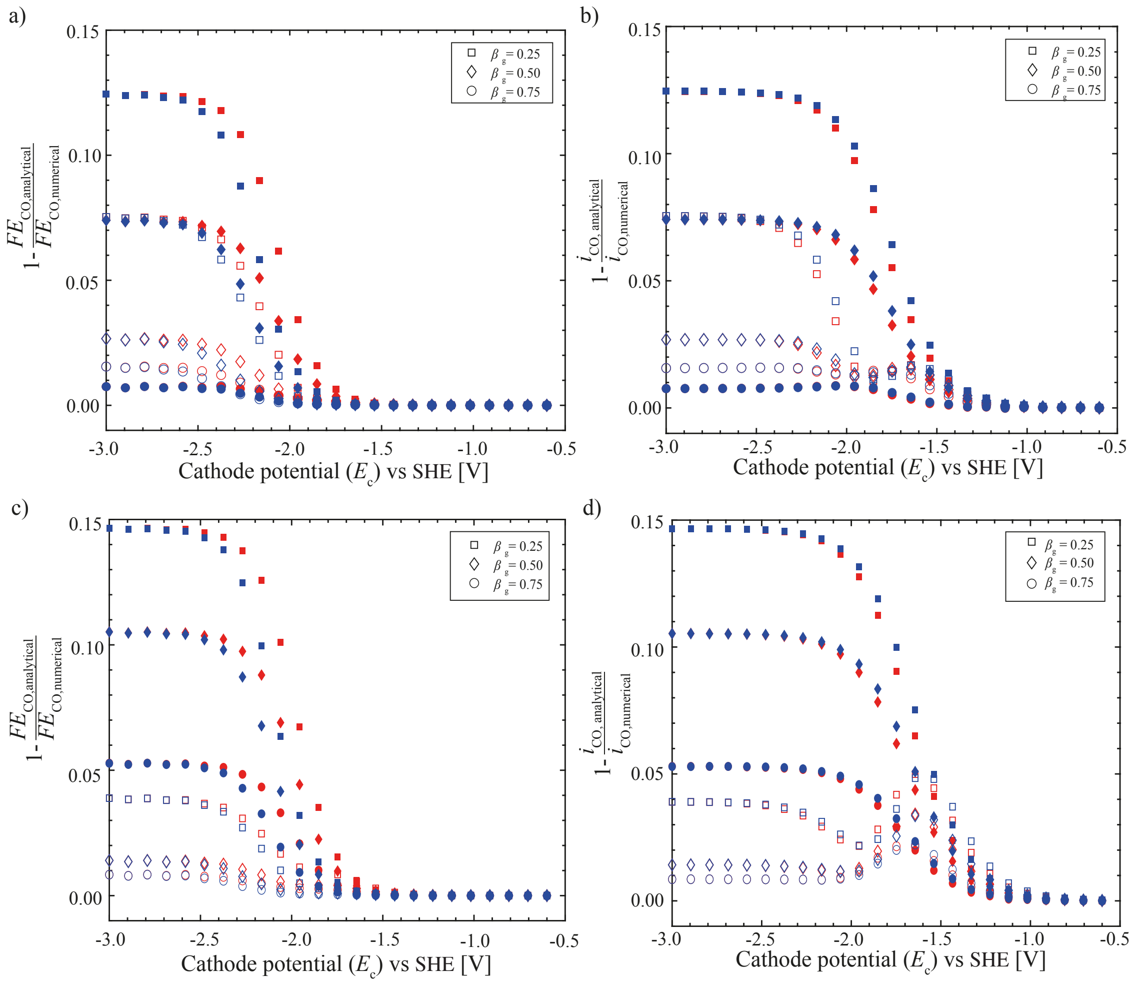

Figure S2: Relative error of analytical predictions towards the numerical results for a tube diameter of $d = 1$ mm (a-b) and of 3 mm (c-d) for three different void fraction β_g. Red corresponding to a pressure of 1 bar and blue to a pressure of 5 bar. Filled symbols $u_B = 0.03\,\mathrm{m\,s^{-1}}$, open symbols $u_B = 0.1\,\mathrm{m\,s^{-1}}$.

4 Fluid/electrochemical properties and model description

4.1 Fluid/electrochemical properties and symbol list

All fluid/electrochemical properties are summarized in Table S2.

Table S2: List of parameters.

Description	Value	Unit	Reference
Electrolyte (1M $KHCO_3$)			
μ ($T = 20\,°C$, $p = 1\,bar$)	$1001.6 \cdot 10^{-6}$	Pa s	[7]
ρ ($T = 20\,°C$, $p = 1\,bar$)	998.21	$kg\,m^{-3}$	[7]
γ ($T = 20\,°C$)	72.74	$N\,m^{-1}$	[7]
D_{CO_2} ($T = 25\,°C$)	$1.97 \cdot 10^{-9}$	$m^2\,s^{-1}$	[8]
D_{HCO_3-} ($T = 25\,°C$)	$1.185 \cdot 10^{-9}$	$m^2\,s^{-1}$	[9]
$D_{CO_3^{2-}}$ ($T = 25\,°C$)	$9.23 \cdot 10^{-10}$	$m^2\,s^{-1}$	[9]
D_{OH} ($T = 25\,°C$)	$5.293 \cdot 10^{-9}$	$m^2\,s^{-1}$	[9]
H^{cp} ($T = 25\,°C$)	$3.4 \cdot 10^{-4}$	$mol\,m^{-3}\,Pa^{-1}$	[10]
Electrochemical reaction			
$i_{0,COER}$	0.1098	$A\,m^{-2}$	[11]
α_{COER}	0.17	-	[11]
E^0_{COER}	-0.11	V	[11]
$i_{0,HER}$	$4.1439 \cdot 10^{-5}$	$A\,m^{-2}$	[11]
α_{HER}	0.25	-	[11]
E^0_{HER}	0	V	[11]
Homogeneous reaction			
k_{1f}	2.23	$mol^{-1}\,s$	[12]
k_{1r}	$1.5494 \cdot 10^{-5}$	s^{-1}	[13]
k_{2f}	$6 \cdot 10^{6}$	$mol^{-1}\,s$	[12]
k_{2r}	$4.6821 \cdot 10^{4}$	s^{-1}	[13]
Unit cell geometry			
d	1 - 3	mm	[14]
u_B	0.01 - 0.3	$m\,s^{-1}$	[15]
β_g	0.25 - 0.75	-	
p	1 - 5	bar	[16]

4.2 Taylor flow features

Taylor flow is introduced in the cathode chamber of a tubular zero-gap CO_2 electrolyser. The cathode flow channel in this study is modelled through a unit cell approach with the assumption of axisymmetry and steady-state. It is assumed that due to the periodicity of the flow, a single gas bubble surrounded by a liquid film and separated by two liquid half-slugs on either side, called a unit cell (UC), can be considered a representative of the flow [17]. The bubble's diameter can be determined based on the thickness of the lubrication film (δ_F), which forms around the bubble. The bubble (L_B) and slug (L_S) lengths can be obtained from the unit cell length (L_{UC}) and void fraction (β_g). The unit cell length and void fraction depend on the superficial gas and liquid velocities ratio and the cross junction geometry [18, 19]. Therefore, unit cell length and void fraction are directly used as input parameters to arrive at the modelling domain in this study. The unit cell length is given based on the diameter ($L_{UC} = 5d$), while the void fraction gives how much of this unit cell is occupied by gas as the ratio between the gas bubble volume and unit cell volume ($\beta_g = \frac{V_B}{V_{UC}}$). The resulting slug length for each void fraction as well as the geometry properties are summarised in Table S3.

Table S3: Taylor flow features for axisymmetric unit cell geometry.

Description	Definition	Unit
Film thickness [20]	$\delta_F = d\frac{0.66Ca^{2/3}}{1+3.33Ca^{2/3}}$	m
Bubble diameter	$d_B = d - 2\delta_F$	m
Film length	$L_F = \frac{d^2}{d_B^2}\beta_g L_{UC} - \frac{2}{3}d_B$	m
Bubble length	$L_B = L_F + d_B$	m
Slug length	$L_S = L_{UC} - L_B$	m

4.3 Governing equations

The unit cell is described by a bubble moving in a round channel with the diameter d. The modelling domain is, therefore, two dimensional and axisymmetric. Only the liquid phase is modelled, and the bubble shape is assumed to remain constant based on the steady-state unit cell approach, which represents a bubble at a fixed location in the reactor. Nonetheless a change in bubble length and bubble velocity is expected based on the diffusion of CO_2 to the reaction side, comparable to the process of dissolution. However, as in this study the reaction products H_2 and CO are formed, which have a low solubility in the liquid electrolyte and are therefore expected to form gas bubbles, it is not evident how the bubble size and composition change over the reactor length. The single-phase flow is described by solving the incompressible Navier-Stokes equation for laminar flow. Mass transfer is modelled by

solving the advection-diffusion equation. The dimensionless governing equations where we use a * for all dimensionless quantities are summarised in Table S4. They were obtained by dividing all lengths with the channel diameter d, and all velocities with the liquid phase velocity which is determined under the assumption of a stagnant film [6] $\frac{u_{\mathrm{TP}}}{u_{\mathrm{B}}} = 1 - \frac{4\delta_{\mathrm{F}}}{d}$. All concentrations are scaled with the saturation concentration of CO_2 (c_{CO_2}). These choices lead to the normalization of the pressure by dividing it by $\mu u_{\mathrm{TP}}/d$ and the introduction of the Reynolds number $Re = \rho u_{\mathrm{TP}} D/\mu$ and Peclét number $Pe = ud/D$ in the governing equations.

Table S4: Dimensionless governing equations.

Mass conservation
$\frac{1}{r^*}\frac{\partial}{\partial r^*}\left(r^* u_r^*\right) + \frac{\partial u_z^*}{\partial z^*} = 0$
**Momentum conservation**
$Re\left[u^*.\nabla^* u^*\right]_r = -\frac{\partial p^*}{\partial r^*} + \left[\nabla^{2*} u^*\right]_r$
$Re\left[u^*.\nabla^* u^*\right]_z = -\frac{\partial p^*}{\partial z^*} + \left[\nabla^{2*} u^*\right]_z$
**Species conservation**
$Pe\left[u^*.\nabla^* c_i^*\right]_r = \left[\nabla^{2*} c_i^*\right]_r$
$Pe\left[u^*.\nabla^* c_i^*\right]_z = \left[\nabla^{2*} c_i^*\right]_z$

4.4 Boundary conditions and electrochemical model

The simulations are taken in the reference frame of the bubble, therefore, the bubble velocity is set as moving wall, and the Hagen-Poiseuille parabolic velocity profile describes the inflow and outflow [21]. A zero slip condition is applied at the interface of the bubble. For the species transport, the interface between bubble and liquid is treated as a free surface, and the saturation concentration, $c^*_{CO_2}$ is obtained from Henry's law ($c^*_{CO_2} = H_{\mathrm{i}} P_{\mathrm{B}}$) assuming constant pressure inside the bubble. The concentration is then corrected for the electrolyte salinity with the Sechenov equation [22]. At the inlet and outlet, the periodic boundary condition assumes that the concentration and fluxes of species are the same [23, 24]. Finally, to complete the model, an electrochemical wall reaction is imposed. The boundary conditions are summarized in Table S5.

The surface reaction rate, $R_{\mathrm{i}} = \frac{\nu i_{\mathrm{i}}}{zF}$, is determined based on the electrochemical Butler-Volmer reaction kinetics. It is assumed that the anodic exponential term becomes negligible compared to the cathodic term at herein studied cathode potentials. This gives the partial current density i_{CO} for the reduction of CO_2 to CO as the concentration-dependent Butler-

Table S5: Boundary conditions for the numerical unit cell model, with $\hat{z}$ denoting the unit vector in the z direction in similarly in r direction for $\hat{r}$.

**Boundary**	**Hydrodynamics**	**Species Transport**
**Inlet**	$u^* = \left(2\left(1-4r^{*2}\right) - \frac{u_B}{u_{TP}}\right)\hat{z} + 0\hat{r}$	Periodic Boundary Condition
**Outlet**	$u^* = \left(2\left(1-4r^{*2}\right) - \frac{u_B}{u_{TP}}\right)\hat{z} + 0\hat{r}, P^* = 0$	Periodic Boundary Condition
**Interface**	Slip condition, $\frac{\partial u^*}{\partial n^*} = 0$ - Free surface	$c_i^* = \frac{c_i^0}{c_{CO_2}^0}$
**Wall**	No slip, $u^* = -\frac{u_B}{u_{TP}}\hat{z} + 0\hat{r}$	$\nabla_n^* c_i^* = \frac{R_i(\eta)d}{D_{CO_2}c_{CO_2}^0}$

Volmer equation

$$i_{CO} = -i_{0,COER}\frac{c_{CO_2,wall}}{c_{ref,CO_2}}\exp\left(\frac{-\alpha_{COER}F}{RT}\eta_{COER}\right), \tag{S7}$$

while the competing hydrogen evolution reaction (HER) is mass transfer independent (see Eq. 10). The kinetic constants like exchange current density $i_{0,COER}$, cathodic transfer coefficient α_{COER}, and reference concentration c_{ref,CO_2} are taken from the study by Wu [11] and are summarized in Table S2.

5 Validation

5.1 Hydrodynamics

For Taylor flow in circular capillaries, only a small portion of the liquid is transported through the film, while the rest of the fluid circulates between the bubbles creating the well-known vortices[25]. The vortex centre is the position at which the velocity in the liquid slugs becomes zero. Thulasidas et al. [26] derived the position of the vortex centre from the Hagen-Poiseullie parabolic velocity profile inside the liquid slug

$$r_0 = \frac{d}{2\sqrt{2}}\sqrt{2 - \frac{|u_{\mathrm{B}}|}{|u_{\mathrm{TP}}|}}. \tag{S8}$$

They further conducted experiments to determine the vortex centre as a function of capillary number. The velocity profile inside the liquid slug is validated by comparing the simulation results with the experimental work of Thulasidas et al. [26] and the analytical expression in Figure S3. The velocity profile in the film is validated against the theoretical model developed by Abiev [27]. The theoretical model is solved in Matlab to determine the velocity profile inside the liquid film and compared with the hydrodynamic simulations in COMSOL. For this a diameter of 3 mm and a velocity of 0.3 m s^{-1} is set. Figure S3 shows the expected trend of the plug flow behaviour for the theoretical velocity profile, while the numerical simulation slightly deviates from this. This is similar to the results by Martínez et al.[24], while the error remains below 6 %.

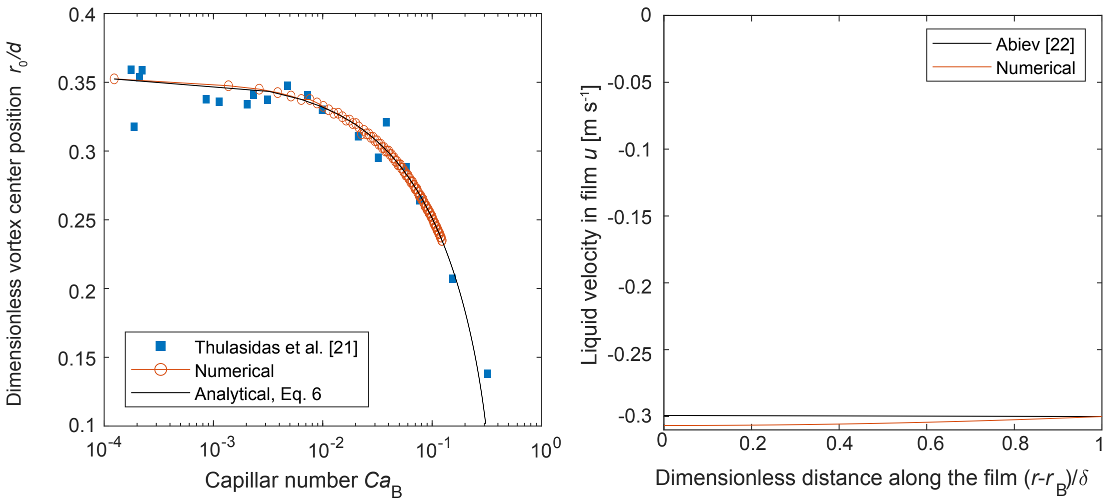

Figure S3: Vortex center position (r_0) as a function of the capillary number (left) and velocity profile in the film region (right).

5.2 Mass transfer

The mass transfer coefficient over the entire bubble in the unit cell is compared to the results from Martïnez et al. [24]. The unit cell geometry is based on the reference case in their work, and the mesh is obtained with a fluidics controlled mesh in COMSOL. The results in terms of mesh, surface area and mass transfer coefficient are shown in Table S6. An error of less than 2 % is found in the calculation of the mass transfer coefficient. This deviation can be attributed to the difference in the mesh.

Table S6: Comparison mass transfer

	Martínez [[24]]	This work
Total mesh elements	77 318	52 724
Elements in liquid film	19	22
Surface are (Unit cell)	$7.50 \cdot 10^{-5}$ [m^2]	$7.50 \cdot 10^{-5}$ [m^2]
$k_L a$ (Unit cell)	0.080 [m$^3{}_L$/m$^3{}_{UC}$/s]	0.081 [m$^3{}_L$/m$^3{}_{UC}$/s]

5.3 Buffer reaction

The homogeneous carbon equilibrium reaction occurs in the liquid electrolyte as soon as carbon dioxide is introduced into the solution. With increasing, production of hydroxide ions from the electrochemical reaction CO_2 starts reacting in a buffer reaction. The following steps commonly describe the buffer reactions

$$CO_2 + OH^- \underset{-\,k_{1r}}{\overset{+\,k_{1f}}{\rightleftharpoons}} HCO_3^- \tag{S9}$$

$$HCO_3^- + OH^- \underset{-\,k_{2r}}{\overset{+\,k_{2f}}{\rightleftharpoons}} CO_3^{2-} \tag{S10}$$

The forward reaction rates are summarized in Table S2, while the backward reaction rates are corrected for the electrolyte salinity [13]. For H-cell and GDE reactors, the species balance is solved in addition to the homogenous reaction as a source term, while migration is neglected [28–31]. In this work, the transfer of hydroxide ions across the membrane, including migration, is not explicitly modelled to reduce computational costs. Further, as the homogenous reactions lead to a stiff non-linear system of equations, the computational time and mesh requirements increase drastically. Therefore, the unit cell's geometry is simplified according to the analytical model; the film and slug region are viewed independent and the diffusion layer thickness is set to the film thickness ($\delta = \delta_F$). This leads to a 1D reaction-diffusion system with the saturation concentration and slug concentration as the boundary condition respectively for the two regions. In Figure S4 the current density for different electrolyte compositions and cases is displayed. No reaction, in this case, refers to the assumption that the flux of hydroxide ions across the membrane is equal to the production rate at the electrode leading to a current independent pH near the catalyst. Reaction refers to the other extreme case assuming no flux of hydroxide ions across the walls. It becomes evident that for low cathode potentials, the effect of the buffer reaction can be neglected, while for higher potentials,

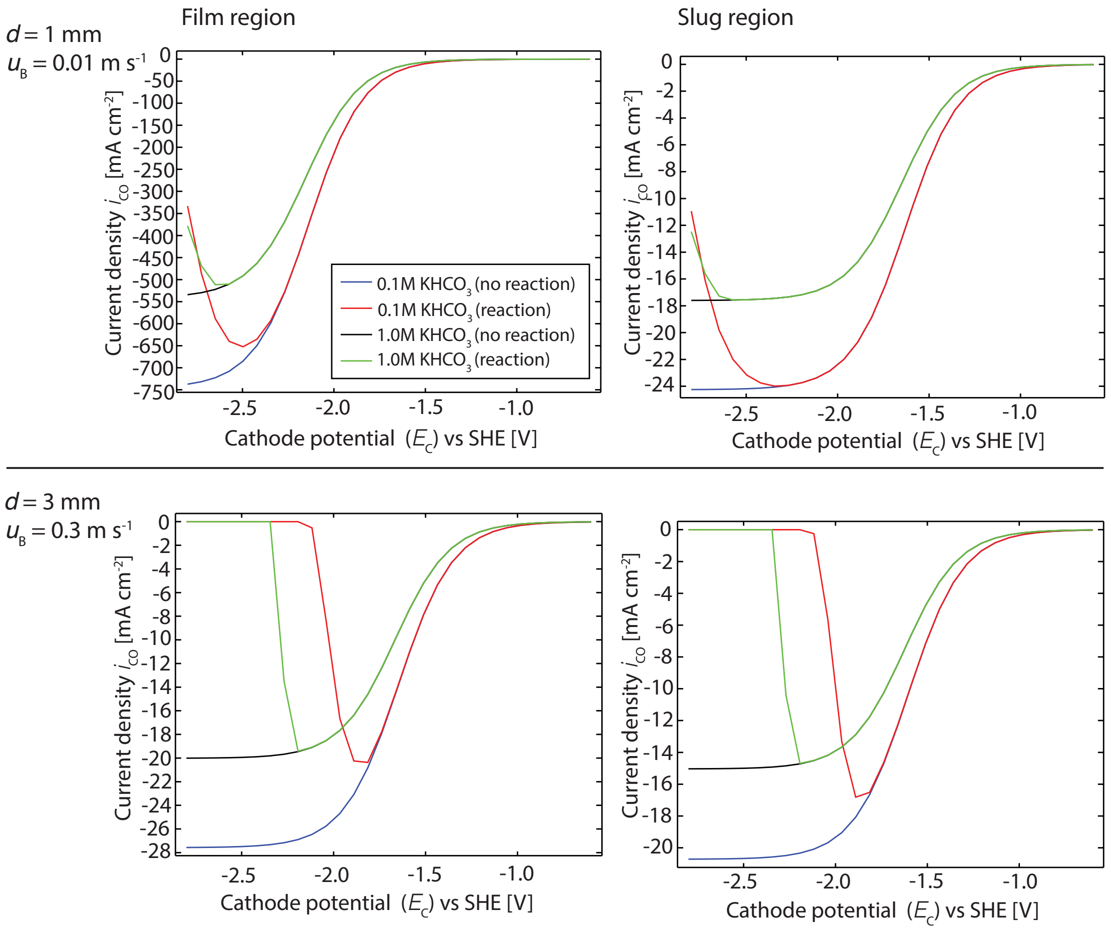

Figure S4: Current density over cathode potential from 1D diffusion reaction model with $\delta = \delta_F$.

the buffer reaction leads to a decrease in current density. The onset of this effect depends on the concentration of the electrolyte. As the 1 M $KHCO_3$ electrolyte shows a more significant buffer capacity and the depletion of CO_2 in the homogenous reaction starts setting in at higher potentials and values close to the theoretical limiting current density, we choose in this work an electrolyte of 1 M $KHCO_3$ and work under the assumption that flux across the membrane equals the production rate. Therefore, the homogenous buffer reaction is not modelled explicitly, and the potential window is chosen between -0.6 to -2.6 V vs SHE.

6 Mesh features and numerical solver

A user-controlled mesh with unstructured quadrilateral elements is built, and a boundary layer with controlled element size and growth rate is introduced at the bubble's interface and wall. All meshes guarantee a minimum of 5 elements in the film region for all film thicknesses [32]. The mesh is then optimized to capture all relevant parameter combinations, hydrodynamics and species transport. The different meshes studied are summarized in Table S7. Note that the mesh parameters depend on the bubble velocity u_B and channel diameter D. For this study, the lower and upper limits of velocities and diameter are chosen for the mesh study. Relative tolerances of $1 \cdot 10^{-3}$ and $5 \cdot 10^{-4}$ are set for achieving convergence for the velocity profile and species transport, respectively. For hydrodynamics, the velocity profiles inside liquid slugs are observed. In the vortex centre, zero velocity is expected; therefore, the change in velocity at the vortex centre is taken as a point of comparison for the accuracy of the mesh, as shown in Figure S6. For the species transport, the local current density at the cathode over the unit cell and the concentration profile of CO_2 in the liquid slug is observed (Figure S6). Based on this, Mesh D is chosen for all simulations in this work. The mesh is shown in Figure S5.

Note: All simulations for the mesh independence study are performed on a computer with an Intel Core i5-4690 processor, 3.5GHz processor speed, and 24GB RAM. All other simulations are carried out on a cluster with a varying allocation of nodes.

Table S7: Mesh details for mesh independence study.

	Total elements	biggest element (µm)	smallest element (µm)	Elements in film	Computational effort (seconds)	u(r_0) $\mathrm{cm\,s^{-1}}$	$i_{Lim,CO}$ $\mathrm{mA\,cm^{-2}}$
Case A							
(1)	17 291	24 788	3 615	11	23	0.24	28.75
(2)	59 516	25 255	382	11	43	$3.28 \cdot 10^{-3}$	217.26
Case B							
(1)	60 123	10 210	2 869	11	78	0.19	24.13
(2)	121 839	15 923	182	11	79	$2.99 \cdot 10^{-3}$	217.42
Case C							
(1)	69 315	10 210	2 869	19	96	0.19	21.75
(2)	161 335	15 923	142	19	103	$2.99 \cdot 10^{-3}$	217.42
**Case D**							
**(1)**	**73 911**	**10 210**	**2 869**	**24**	**119**	**0.19**	**19.04**
**(2)**	**181 083**	**15 923**	**96**	**24**	**117**	$\mathbf{2.99 \cdot 10^{-3}}$	**217.40**
Case E							
(1)	258 876	5 105	1 094	30	2 238	0.19	17.6
(2)	566 412	8 158	53	30	2 522	$2.18 \cdot 10^{-3}$	217.60
Case F							
(1)	277 260	5 105	1 090	38	2 376	0.18	18.30
(2)	645 342	7 962	63	38	3 465	$2.19 \cdot 10^{-3}$	217.61

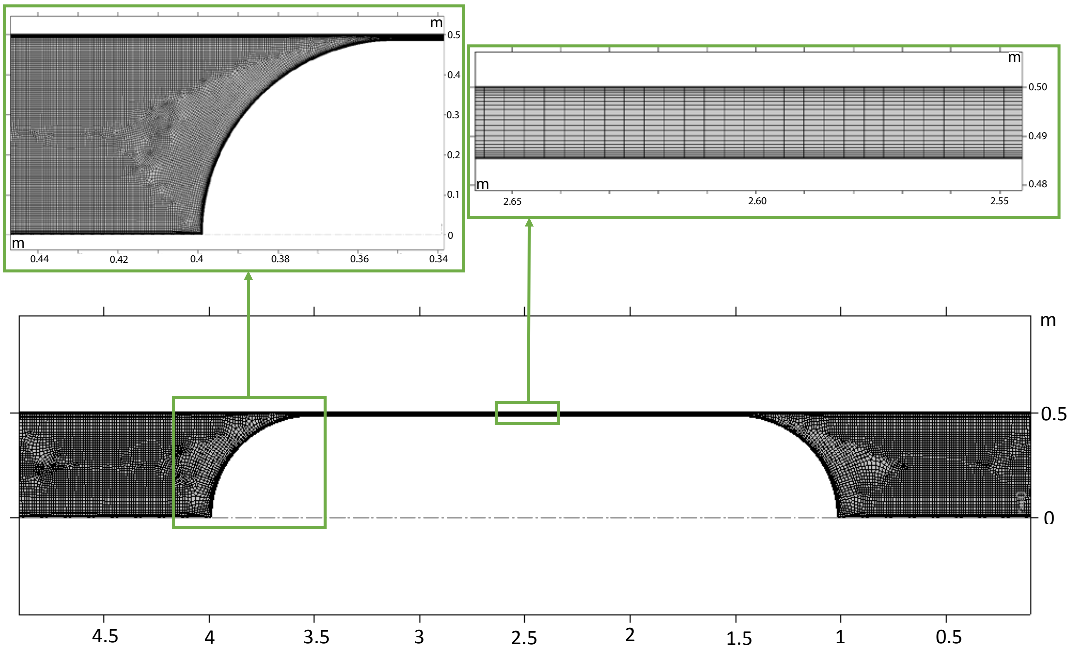

Figure S5: Mesh of the liquid domain in the unit cell with zoomed-in regions on bubble cap and film.

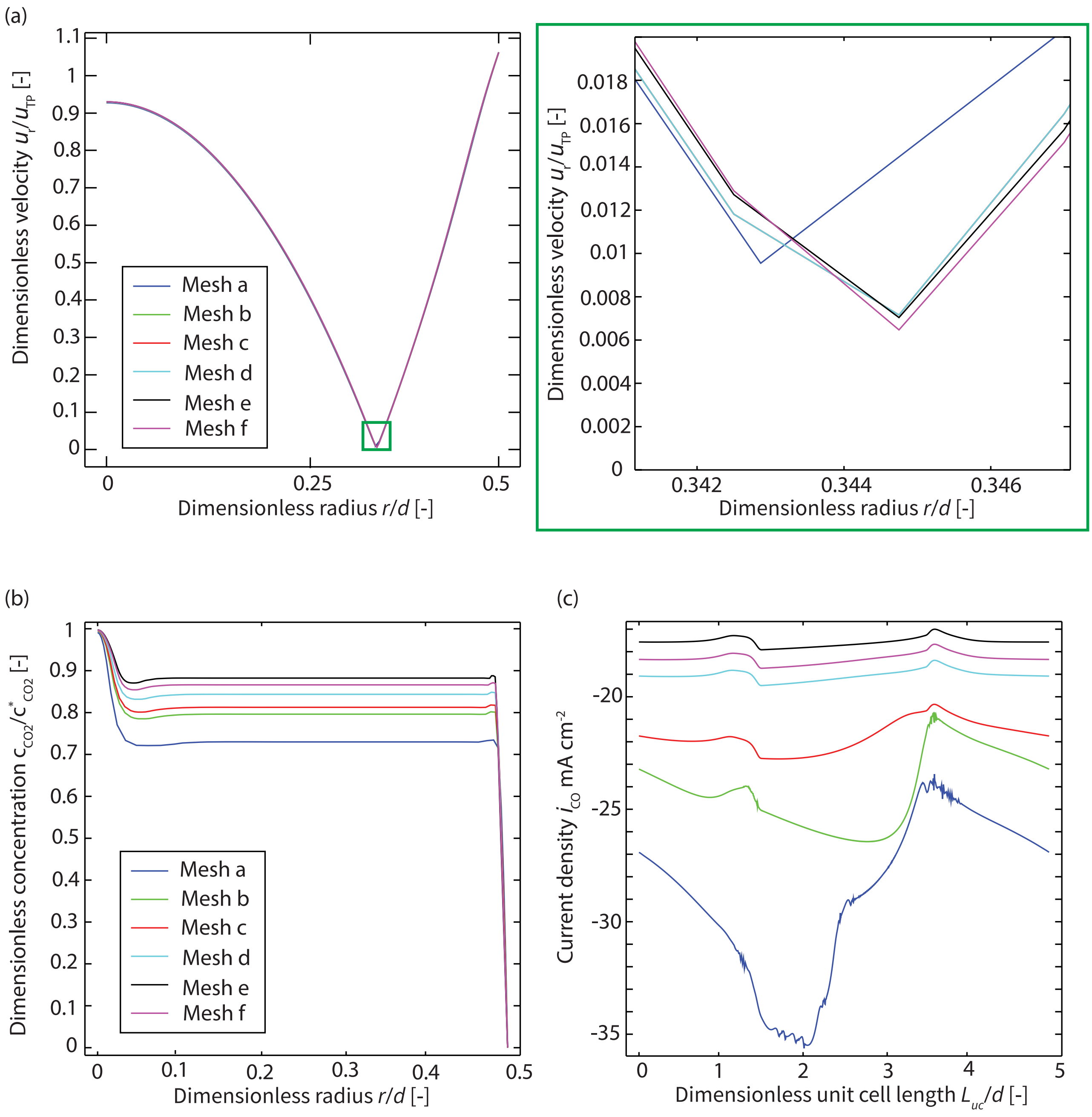

Figure S6: Velocity profile in the liquid slug for different meshes with a zoomed-in view of the vortex centre for a capillary with d = 3 mm and a bubble velocity of 0.3 m s^{-1} (a). CO_2 concentration profile (b) and local current density (c) for different meshes for a capillary with d = 3 mm and a bubble velocity of 0.3 m s^{-1}.

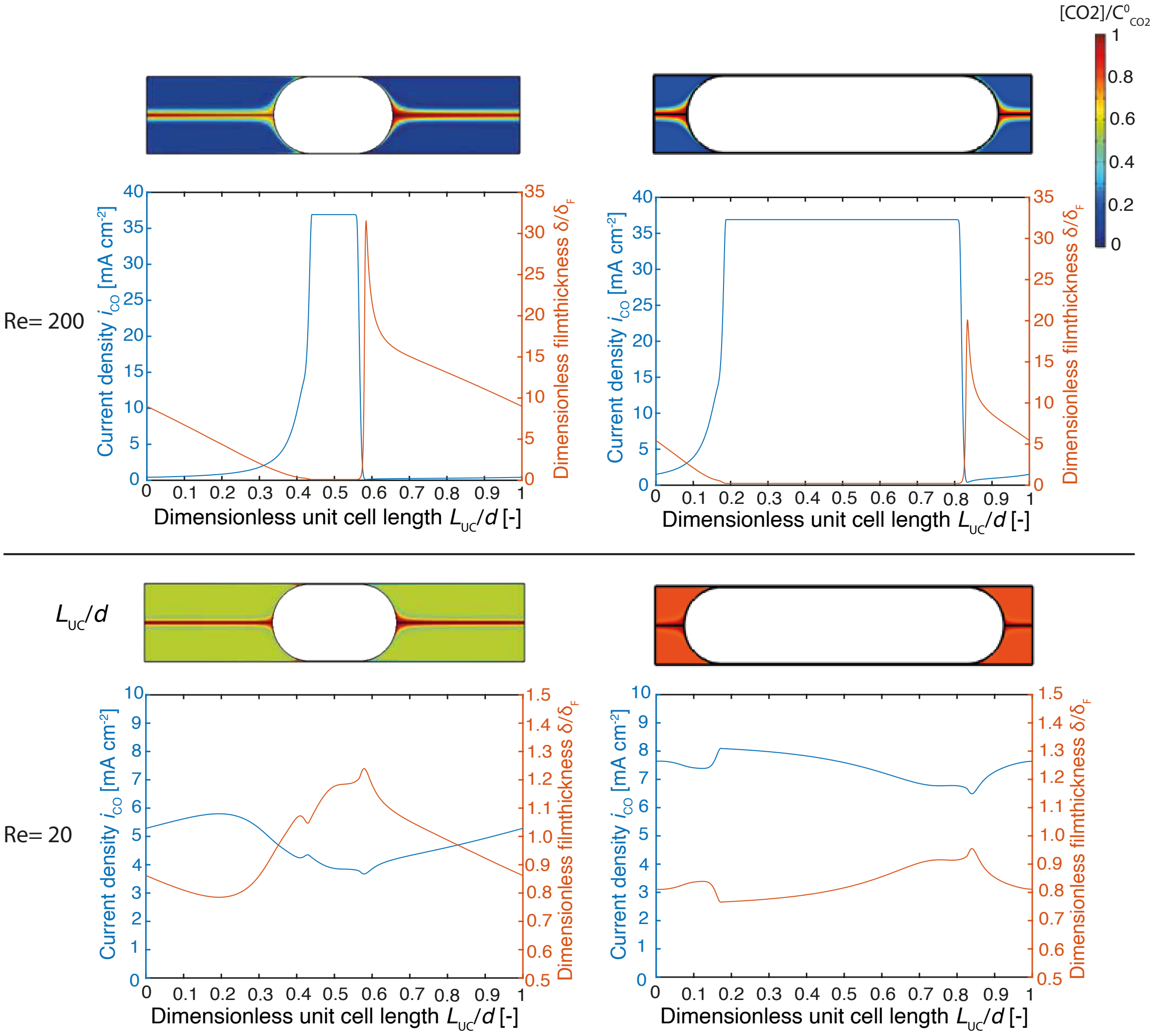

Figure S7: Dimensionless concentration of CO_2 for different void fractions and Reynolds-numbers with the limiting current density over the unit cell length and the dimensionless diffusion layer thickness.

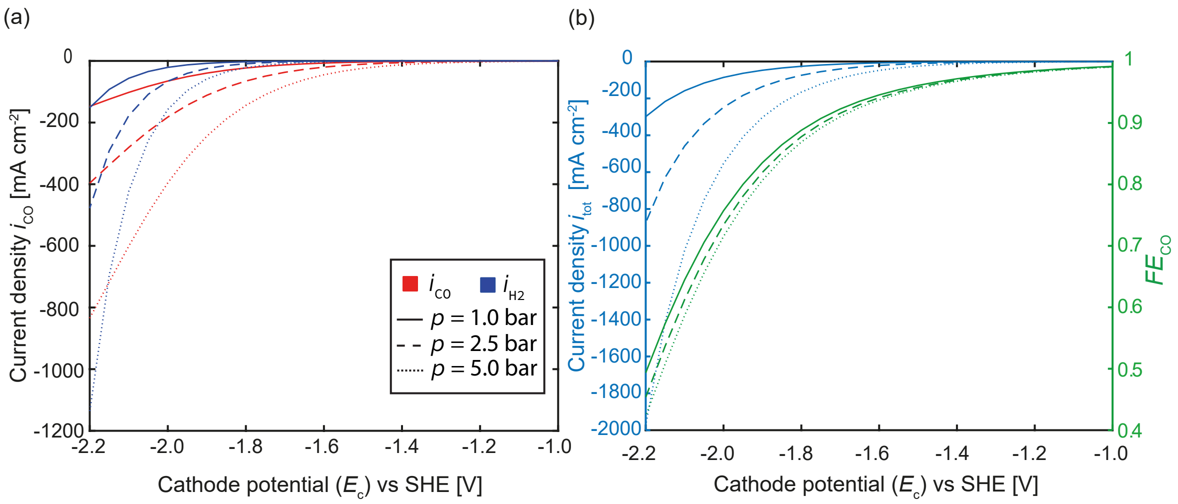

Figure S8: The influence of pressure on the partial current densities (a), and the sum of the partial current density and faradaic efficiency (b). Pressure 1 bar solid line, 2.5 bar dashed and 5 bar doted lines.

References

(1) Lobaccaro, P.; Singh, M. R.; Clark, E. L.; Kwon, Y.; Bell, A. T.; Ager, J. W. Effects of temperature and gas–liquid mass transfer on the operation of small electrochemical cells for the quantitative evaluation of CO_2 reduction electrocatalysts. *Phys. Chem. Chem. Phys.* **2016**, *18*, 26777–26785, DOI: 10.1039/C6CP05287H.

(2) Ressel, S.; Laube, A.; Fischer, S.; Chica, A.; Flower, T.; Struckmann, T. Performance of a vanadium redox flow battery with tubular cell design. *Journal of Power Sources* **2017**, *355*, 199–205, DOI: 10.1016/j.jpowsour.2017.04.066.

(3) Kibria, M. G.; Edwards, J. P.; Gabardo, C. M.; Dinh, C.-T.; Seifitokaldani, A.; Sinton, D.; Sargent, E. H. Electrochemical CO_2 reduction into chemical feedstocks: From mechanistic electrocatalysis models to system design. *Advanced Materials* **2019**, *31*, 1807166, DOI: 10.1002/adma.201807166.

(4) Laube, A.; Hofer, A.; Sánchez Batalla, B.; Ressel, S.; Chica, A.; Fischer, S.; Weidlich, C.; Bachmann, J.; Struckmann, T. Tubular membrane electrode assembly for PEM electrolysis. *International Journal of Hydrogen Energy* **2022**, *47*, 15943–15951, DOI: 10.1016/j.ijhydene.2022.03.135.

(5) Dinh, C.-T.; Burdyny, T.; Kibria, M. G.; Seifitokaldani, A.; Gabardo, C. M.; de Arquer, F. P. G.; Kiani, A.; Edwards, J. P.; Luna, P. D.; Bushuyev, O. S.; Zou, C.; Quintero-Bermudez, R.; Pang, Y.; Sinton, D.; Sargent, E. H. CO_2 electroreduction to ethylene via hydroxide-mediated copper catalysis at an abrupt interface. *Science* **2018**, *360*, 783–787, DOI: 10.1126/science.aas9100.

(6) Kreutzer, M. T.; Kapteijn, F.; Moulijn, J. A.; Heiszwolf, J. J. Multiphase monolith reactors: Chemical reaction engineering of segmented flow in microchannels. *Chemical Engineering Science* **2005**, *60*, 5895–5916, DOI: 10.1016/j.ces.2005.03.022.

(7) *VDI-Wärmeatlas*, *Stoffwerte von Wasser*; Springer Berlin Heidelberg: Berlin, Heidelberg, 2006, pp 133–147, DOI: 10.1007/978-3-540-32218-4_11.

(8) Frank, M. J. W.; Kuipers, J. A. M.; van Swaaij, W. P. M. Diffusion coefficients and viscosities of CO_2 + H_2O, CO_2 + CH_3OH, NH_3 + H_2O, and NH_3 + CH_3OH liquid mixtures. *Journal of Chemical & Engineering Data* **1996**, *41*, 297–302, DOI: 10.1021/je950157k.

(9) Newmann, J. S.; Thomas-Alyea, K. E., *Electrochemical systems*, 3rd ed.; Hoboken, N.J. : J. Wiley: 2012.

(10) Sander, R. Compilation of Henry's law constants (version 4.0) for water as solvent. *Atmospheric Chemistry and Physics* **2015**, *15*, 4399–4981, DOI: 10.5194/acp-15-4399-2015.

(11) Wu, K.; Birgersson, E.; Kim, B.; Kenis, P. J. A.; Karimi, I. A. Modeling and experimental validation of electrochemical reduction of CO_2 to CO in a microfluidic cell. *Journal of The Electrochemical Society* **2014**, *162*, F23–F32, DOI: 10.1149/2.1021414jes.

(12) Schulz, K.; Riebesell, U.; Rost, B.; Thoms, S.; Zeebe, R. Determination of the rate constants for the carbon dioxide to bicarbonate inter-conversion in pH-buffered seawater systems. *Marine Chemistry* **2006**, *100*, 53–65, DOI: 10.1016/j.marchem.2005.11.001.

(13) Millero, F. J.; Graham, T. B.; Huang, F.; Bustos-Serrano, H.; Pierrot, D. Dissociation constants of carbonic acid in seawater as a function of salinity and temperature. *Marine Chemistry* **2006**, *100*, 80–94, DOI: 10.1016/j.marchem.2005.12.001.

(14) Olesen, A. C.; Frensch, S. H.; Kær, S. K. Towards uniformly distributed heat, mass and charge: A flow field design study for high pressure and high current density operation of PEM electrolysis cells. *Electrochimica Acta* **2019**, *293*, 476–495, DOI: 10.1016/j.electacta.2018.10.008.

(15) Berčič, G.; Pintar, A. The role of gas bubbles and liquid slug lengths on mass transport in the Taylor flow through capillaries. *Chemical Engineering Science* **1997**, *52*, 3709–3719, DOI: 10.1016/S0009-2509(97)00217-0.

(16) Morrison, A. R. T.; van Beusekom, V.; Ramdin, M.; van den Broeke, L. J. P.; Vlugt, T. J. H.; de Jong, W. Modeling the electrochemical conversion of carbon dioxide to formic acid or formate at elevated pressures. **2019**, *166*, E77–E86, DOI: 10.1149/2.0121904jes.

(17) Edvinsson, R. K.; Irandoust, S. Finite-element analysis of Taylor flow. *AIChE Journal* **1996**, *42*, 1815–1823, DOI: 10.1002/aic.690420703.

(18) Shao, N.; Salman, W.; Gavriilidis, A.; Angeli, P. CFD simulations of the effect of inlet conditions on Taylor flow formation. *International Journal of Heat and Fluid Flow* **2008**, *29*, 1603–1611, DOI: 10.1016/j.ijheatfluidflow.2008.06.010.

(19) Van Steijn, V.; Kleijn, C. R.; Kreutzer, M. T. Predictive model for the size of bubbles and droplets created in microfluidic T-junctions. *Lab on a Chip* **2010**, *10*, 2513–2518, DOI: 10.1039/C002625E.

(20) Aussillous, P.; Quéré, D. Quick deposition of a fluid on the wall of a tube. *Physics of Fluids* **2000**, *12*, 2367–2371, DOI: 10.1063/1.1289396.

(21) Kreutzer, M. T.; Kapteijn, F.; Moulijn, J. A.; Kleijn, C. R.; Heiszwolf, J. J. Inertial and interfacial effects on pressure drop of Taylor flow in capillaries. *AIChE Journal* **2005**, *51*, 2428–2440, DOI: 10.1002/aic.10495.

(22) Weisenberger, S.; Schumpe, A. Estimation of gas solubilities in salt solutions at temperatures from 273 K to 363 K. *AIChE Journal* **1996**, *42*, 298–300, DOI: 10.1002/aic.690420130.

(23) van Baten, J.; Krishna, R. CFD simulations of wall mass transfer for Taylor flow in circular capillaries. *Chemical Engineering Science* **2005**, *60*, 1117–1126, DOI: 10.1016/j.ces.2004.10.001.

(24) Martínez, F. L. D.; Julcour, C.; Billet, A.-M.; Larachi, F. Modelling and simulations of a monolith reactor for three-phase hydrogenation reactions — Rules and recommendations for mass transfer analysis. *Catalysis Today* **2016**, *273*, 121–130, DOI: 10.1016/j.cattod.2016.04.009.

(25) Taylor, G. I. Deposition of a viscous fluid on a plane surface. *Journal of Fluid Mechanics* **1960**, *9*, 218–224, DOI: 10.1017/S0022112060001055.

(26) Thulasidas, T.; Abraham, M.; Cerro, R. Bubble-train flow in capillaries of circular and square cross section. *Chemical Engineering Science* **1995**, *50*, 183–199, DOI: 10.1016/0009-2509(94)00225-G.

(27) Abiev, R. S. Bubbles velocity, Taylor circulation rate and mass transfer model for slug flow in milli- and microchannels. *Chemical Engineering Journal* **2013**, *227*, 66–79, DOI: 10.1016/j.cej.2012.10.009.

(28) Gupta, N.; Gattrell, M.; MacDougall, B. Calculation for the cathode surface concentrations in the electrochemical reduction of CO_2 in $KHCO_3$ solutions. *Journal of Applied Electrochemistry* **2006**, *36*, 161–172, DOI: 10.1007/s10800-005-9058-y.

(29) Weng, L.-C.; Bell, A. T.; Weber, A. Z. Modeling gas-diffusion electrodes for CO_2 reduction. *Phys. Chem. Chem. Phys.* **2018**, *20*, 16973–16984, DOI: 10.1039/C8CP01319E.

(30) Burdyny, T.; Smith, W. A. CO_2 reduction on gas-diffusion electrodes and why catalytic performance must be assessed at commercially-relevant conditions. *Energy Environ. Sci.* **2019**, *12*, 1442–1453, DOI: 10.1039/C8EE03134G.

(31) Kas, R.; Star, A. G.; Yang, K.; Van Cleve, T.; Neyerlin, K. C.; Smith, W. A. Along the channel gradients impact on the apatioactivity of gas diffusion electrodes at high conversions during CO_2 electroreduction. *ACS Sustainable Chemistry & Engineering* **2021**, *9*, 1286–1296, DOI: 10.1021/acssuschemeng.0c07694.

(32) Gupta, R.; Fletcher, D. F.; Haynes, B. S. On the CFD modelling of Taylor flow in microchannels. *Chemical Engineering Science* **2009**, *64*, 2941–2950, DOI: 10.1016/j.ces.2009.03.018.
